# Approach to Posture and Gait in Huntington’s Disease

**DOI:** 10.3389/fbioe.2021.668699

**Published:** 2021-07-27

**Authors:** Lauren S. Talman, Amie L. Hiller

**Affiliations:** ^1^Department of Neurology, Oregon Health & Science University, Portland, OR, United States; ^2^Portland VA Healthcare System, Portland, OR, United States

**Keywords:** Huntington’s disease, posture, wearable sensors, gait, multi-disciplinary approach

## Abstract

Disturbances of gait occur in all stages of Huntington’s disease (HD) including the premanifest and prodromal stages. Individuals with HD demonstrate the slower speed of gait, shorter stride length, and increased variability of gait parameters as compared to controls; cognitive disturbances in HD often compound these differences. Abnormalities of gait and recurrent falls lead to decreased quality of life for individuals with HD throughout the disease. This scoping review aims to outline the cross-disciplinary approach to gait evaluation in HD and will highlight the utility of objective measures in defining gait abnormalities in this patient population.

## Introduction

Huntington’s disease (HD) is a genetic neurodegenerative disorder caused by autosomal dominant inheritance of an expanded CAG repeat portion in the huntingtin gene on chromosome 4. HD is characterized by progressive motor, cognitive and behavioral changes with “manifest” disease defined by the motor syndrome. “Premanifest” HD describes the entire period prior to the motor onset of disease and “prodromal” HD describes a phase of the premanifest disease when subtle motor symptoms may arise. While striatal degeneration is a pathologic hallmark of HD, longitudinal imaging studies have demonstrated progressive atrophy of the cortex and white matter tracts as well (Tabrizi et al., 2012). There is additional evidence to suggest that degeneration of the cerebellum occurs (Rüb et al., 2013). These pathologic changes help to explain the motor heterogeneity observed clinically in patients with HD. Though adult-onset HD is often recognized by the presence of hyperkinetic movements, namely chorea, individuals with HD also experience impairment involuntary control of movement including bradykinesia, motor impersistence, loss of postural reflexes, and ataxia. This loss of voluntary motor control is often more functionally disabling than the presence of chorea (Hart et al., 2013). Dystonia is common, though the specific effects of dystonia on gait dysfunction are not well established (Vuong et al., 2018). Head-to-head comparisons of gait performance in HD vs. other neurologic diseases are limited, though some studies do suggest that a higher degree of variability in gait measures distinguishes HD from Parkinson’s disease (PD), cerebellar ataxias, and others (Moon et al., 2016). Individuals with HD are at relatively high risk for falls with fallers demonstrating a higher degree of chorea, truncal sway, and bradykinesia as compared to non-fallers (Grimbergen et al., 2008). Abnormalities of posture and gait may arise even in the premanifest stage (Rao et al., 2008). As the disease advances, gait dysfunction can become a significant source of disability and influences overall quality of life (Vuong et al., 2018).

Juvenile HD (JHD), defined as the onset of manifest disease prior to age 20, deserves mention but will not be the focus of this review. Whereas chorea is the most common motor feature of adult-onset HD, JHD typically manifests as an akinetic-rigid syndrome, and large database studies have suggested that gait dysfunction is a more common presenting sign in JHD (Fusilli et al., 2018).

Though there are myriad clinical descriptions of gait in adult-onset HD, quantitative measures of gait dysfunction may provide a more sensitive marker of disease progression. A biomarker is currently lacking in HD, and thus, a noninvasive method of tracking disease and monitoring the effects of intervention is attractive. In this review, wearable sensors will be highlighted as these have significant momentum in the field and have the potential to capture changes in function at home. Due to the complex nature of HD, the focus of this review on gait dysfunction will be presented in the context of a multi-disciplinary model of research and care.

Using a PubMed search with terms including “Huntington’s disease, sensors, gait, posture, multi-disciplinary,” articles presented in this review were selected based on publication date with those published in the prior 10 years prioritized. The purpose of this scoping review is not to present a comprehensive discussion of all available literature but rather to synthesize existing knowledge and highlight the need for future research (Colquhoun et al., 2014).

## Defining Gait Dysfunction in HD Through Use of Wearable Sensors

Clinical evaluation of posture and gait is subjective by nature and rating scales may not reflect motor behavior at home or small changes over time. This includes the Unified Huntington Disease Rating Scale (UHDRS), a tool used to track changes in HD symptoms, with higher scores reflecting greater severity of disease (Huntington Study Group, 1996). The motor portion [total motor score (TMS)] of the UHDRS devotes 12 of 124 points to the examination of gait and balance and, therefore, may be more limited in capturing small changes in these domains over time. In addition to the UHDRS, other clinical scales may correlate with disease severity including Timed Up and Go (TUG), and Berg Balance scale (Rao et al., 2009); however, the Berg Balance Scale may be less sensitive to changes in the premanifest and early manifest stages (Rumpf et al., 2010). Due to possible time and space requirements for the aforementioned scales, researchers have explored several simple clinical tests of balance and found that stance with feet close together and tandem gait tests were sensitive for detecting postural instability (Brožová et al., 2011).

Objective measures of balance and gait using technology have emerged as a more sensitive way to distinguish disease states. Though wearable sensors will be highlighted in this review, there are certainly other objective measures that can be used to assess gait and balance in HD. Studies of posturography have detected impairments in both static and dynamic balance in both premanifest and manifest HD subjects with modest association to disease severity (Reyes et al., 2018). Gait performance using the GAITRite mat may distinguish individuals with HD from controls though may be less sensitive than posturography when these tools are compared head-to-head (Beckmann et al., 2018). Three-dimensional motion capture systems may also be employed as an effective tool to measure the effects of intervention (Mirek et al., 2018).

Wearable inertial sensors such as those utilizing a tri-axial gyroscope, accelerometer, and magnetometer, also objectively measure components of gait and posture. Sensors provide an increased volume of data over longer periods and can be applied in either the laboratory or home setting, the latter of which provides a more accurate reflection of function in daily life. Numerous studies outline the utility of wearable sensors in older adults and individuals with other neurologic disorders such as PD (Rovini et al., 2017). Sensor-based technology in HD is not as well established; however, the use of sensors to objectively define gait parameters in HD could have implications for monitoring disease progression and efficacy of therapeutic interventions.

Several studies have demonstrated that data obtained from sensors may distinguish HD subjects from controls and various stages of HD may be associated with measurable differences in motor activity. Using self-adhesive accelerometer-based sensors in the home setting, one study exploring activity state showed that patients with HD, in general, spend significantly more time lying down as compared to controls (Adams et al., 2017).

There is also evidence to suggest that the severity of motor symptoms may correlate with sensor-based gait measures. In a study of 15 individuals with HD and five controls, accelerometer-based sensors detected between-group differences in deviation step time. Gait parameters differed significantly between those with higher vs. lower TMS (Andrzejewski et al., 2016). In another study that used a single triaxial accelerometer, individuals with HD demonstrated a significant reduction in stride length and gait velocity while stride time and stance time were significantly increased. Measures of gait variability correlated with disease severity as measured by the UHDRS-TMS and total functional capacity (TFC), the latter of which is a surrogate for disease stage (Dalton et al., 2013).

Further exploration into whether wearable sensors may differentiate different disease states supports these earlier findings. In a dual center study, 43 patients with manifest HD and 43 controls completed four 10-m walk tests while wearing accelerometer gyroscope sensors on each shoe. Patients with HD and controls had significantly different stride length, gait velocity, stride time, and stance time. Patients with HD had significantly greater variability in all parameters of gait, with the greatest variability noted in stride time, stance time, and swing time. Disease severity correlated with measures of gait variability with the strongest correlation between these clinical measures and stride time coefficient of variance (CV), swing time CV, and stance time CV. Subgroup analysis of individuals with early, moderate, or advanced disease demonstrated significant between-group differences in stride length, gait velocity, stride time, and stance time with stride time as the sole parameter distinguishing moderate from advanced disease (Gaßner et al., 2020).

The effect of sensory input on postural stability has also been explored using sensors. One observational study of 39 participants including healthy controls, individuals with premanifest and manifest HD demonstrated that those with manifest HD had increased postural sway both in sitting and standing positions (Porciuncula et al., 2020). Postural sway increases for individuals with premanifest HD with the removal of *both* visual sensory input and proprioceptive input while individuals with manifest HD had increased sway with loss of proprioceptive input alone. Gaze fixation appeared to improve sway in the premanifest cohort while it did not affect postural sway in manifest HD. Though further studies are required to confirm this observation, these findings could suggest that early intervention with gaze fixation training in premanifest or early HD may have a beneficial effect on postural sway and balance.

## The Effects of Cognition on Gait in HD

Though disease onset is defined by the emergence of motor symptoms, it is not uncommon for individuals with HD to develop cognitive dysfunction years prior. Cognitive impairment in HD is comprised of a decline in executive function, concentration, and memory (Paulsen, 2011; Teixeira et al., 2016). Furthermore, individuals with HD are known to have difficulty with task shifting and dividing attention (Aron et al., 2003; Vaportzis et al., 2015; Maurage et al., 2017). Formal cognitive testing through neuropsychological evaluation often helps to better define the specific cognitive deficits of an individual.

A link between the decline in cognition and gait dysfunction has been noted in other patient populations (Amboni et al., 2012). Several studies have explored this relationship in HD. A retrospective longitudinal study supports a relationship between mobility as measured by the Tinetti Mobility Test (TMT), measures of cognition, and motor severity as defined by the UHDRS (Kloos et al., 2017). Among the cognitive measures tested, the Symbol Digit Modality Test (SDMT) and Stroop interference correlated more closely than others with measures of mobility, though the strength of this correlation was modest based on applied criteria. The Pearson correlation coefficient was 0.48 and 0.40 for TMT with SDMT and Stroop interference, respectively.

In HD, as in other neurologic disorders, there is decreased automaticity of movement and, thus, attention-requiring tasks often worsen performance. Individuals with HD have difficulty multitasking, and this is highlighted when cognitive interference is imposed during gait evaluation, an approach termed dual-tasking. The decline in the ability to dual-task (DT) has been linked with falls in other populations including older adults, individuals with multiple sclerosis, and those with PD (Toulotte et al., 2006; Hamilton et al., 2009; Jacobs et al., 2014).

Several studies have examined the effect of dual-task conditions on gait performance in HD. One such study used a video motion system to define gait variables in a group of 15 participants with HD and 15 controls. Gait measurements were obtained under motor dual-task conditions (walking while carrying a tray) and cognitive dual-task conditions (walking while counting backward). While gait speed was unchanged with motor DT, this, in addition to cadence and strength, decreased in the setting of cognitive DT in the HD cohort. Decreased gait speed in the cognitive DT condition was associated with an increased TMS on the UHDRS and poorer performance on verbal fluency, Stroop, and the SDMT (Delval et al., 2008).

Another group of researchers aimed to further examine the link between cognition and ability to dual-task and the effect of dual-task performance on fall risk in a group of individuals with manifest HD. Using the walking while talking task (WWTT), both “simple” and “complex” dual-task conditions were explored (Fritz et al., 2016). The dual-task cost (DTC) was defined as the change in performance between dual-task conditions and single-task conditions. The result shows that the time required to complete the simple dual-task WWTT correlated with the TMS, while the complex dual-task time correlated significantly with TFC. Using a patient-reported number of falls over 3 months, the risk of falls did appear to correlate with time for both the simple and complex WWTT tasks; this relationship was particularly strong in the subgroup of individuals with TMS > 35 (more advanced disease).

Additional support for the link between cognitive impairment and the ability to dual-task during measures of balance was brought to light in a 2019 study. A group of 17 individuals with HD and 17 control subjects completed a cognitive battery, motor evaluation, and self-evaluation of balance. Variables of postural sway were measured using the inertial sensor instrumented SWAY (i-SWAY). These variables were measured in multiple conditions: feet together vs. apart, eyes open vs. closed, firm vs. soft surface, with vs. without dual-task (The Controlled Oral Word Association Test). Individuals with HD had higher measures of postural sway (all variables) in all conditions as compared to controls. Narrow base, decreased visual input, and dual-task conditions worsened performance. Impaired visuospatial processing on cognitive measures correlated with increased total sway and jerk. Though postural sway did not correlate with prospective falls, these results may have been limited by self-report (Purcell et al., 2019).

A more recent study from the same group of researchers further explored the effect of dual-task conditions on mobility by comparing gait measures during three 2-min walk tests under single task, fast as possible, and dual-task conditions using the Mobility Lab software and Opal wearable sensors (Opal^TM^, APDM, Inc., Portland, OR, United States). Gait speed, stride length, lateral step variability, and stride length variability differed significantly between HD and control groups under all conditions. Under DT conditions, individuals with HD took a greater number of steps and time to complete a turn (Purcell et al., 2020).

Dual-task training as a strategy to reduce the risk of falls has been noted in older adults and other patient populations (Mirelman et al., 2011; Dorfman et al., 2014; Fritz et al., 2015). Further studies with perhaps less reliance on self-report of falls will be required to determine whether this approach may be applicable and effective for the HD population.

## Exercise and Rehabilitation

Exercise is thought to be a neuroprotective tool in other degenerative disorders (Frazzitta et al., 2015; Paillard et al., 2015); however, the effects of exercise and rehabilitation on the course of HD are still under review. Furthermore, the response of balance and gait to exercise/rehabilitation interventions in HD has not been systematically studied with available studies exploring this topic employing varying interventions and recruiting small sample sizes.

A recent mixed-methods systematic review aimed to provide recommendations based on studies exploring the effects of physical therapy and exercise interventions on overall function in HD (Quinn et al., 2020). While there was only weak evidence for balance training and postural control training, there was strong evidence for gait training in HD, stemming from a review of six randomized control trials (RCTs), six pre/posttest control group studies, and two studies without control groups. Though one of the RCTs did provide accelerometers to record daily activity (Busse et al., 2013), others relied on observation or exercise diaries to track adherence to and effect of the intervention. The challenge of generalizing effects of exercise/therapy is highlighted by the variability of interventions employed in the RCTs included in this review: a combined in-gym bicycling program and home walking program (Busse et al., 2013), a home exercise program guided by an exercise DVD (Khalil et al., 2013), home use of a danced-focused video game (Kloos et al., 2013), one-on-one task-specific therapy at home (Quinn et al., 2014), gym aerobic and resistance training (Quinn et al., 2016), and a multidisciplinary intervention consisting of physical therapy and cognitive therapy along with a home exercise program (Cruickshank et al., 2018). Gait speed and balance (as measured by the Berg Balance Score) improved primarily with the exercise DVD/home walking program. One-on-one physical therapy improved gait and mobility measures including gait speed and TUG (Quinn et al., 2020).

The effects of exercise on cognition in HD are unknown and data are limited. While one aforementioned multimodal exercise intervention did lead to improvement in verbal learning and memory, others have shown no change in cognitive measures pre and post-intervention (Busse et al., 2013; Quinn et al., 2016; Frese et al., 2017; Cruickshank et al., 2018). Larger studies with reproducible protocols will be needed to address this question further.

Several studies have emphasized the utility of more intensive multidisciplinary inpatient rehabilitation programs (Zinzi et al., 2009; Ciancarelli et al., 2013; Thompson et al., 2013). One such Norwegian program demonstrated improved gait function, balance, physical quality of life, and mood when patients with early to mid-stage HD were admitted to an inpatient rehabilitation center for three 3-week sessions per year (Piira et al., 2013, 2014). Though this approach is not feasible in many communities, these studies suggest that more intensive rehabilitation programs incorporating a multidisciplinary approach through physical therapy, occupational therapy, and speech therapy may be the most effective model to improve overall function in HD.

## Discussion

Gait dysfunction in HD is multifactorial, relating not only to the effects of motor manifestations of the disease but also cognitive limitations. Clinical evaluation of gait dysfunction relies on subjective descriptions, while the use of sensor-based technology provides more objective measures. Sensor technology has evolved, with the current focus on wearable devices which provide real-time data in both the laboratory and home settings. Sensors appear to be sensitive in distinguishing between individuals with HD and controls, and sensor-based gait parameters may correlate with disease stage and motor severity. Individuals with HD have decreased reserve to adapt to cognitive dual tasking with worsened performance on measures of both postural sway and gait. The correlation between performance on cognitive dual tasks and risk of falls is yet to be determined due to reliance on self-report. Early identification of individuals with executive dysfunction through formal neuropsychiatric cognitive testing may help clinicians to target particular patients for dual-task training or exercise interventions. Furthermore, particular attention to individuals with premanifest HD may allow for earlier intervention.

There are clear limitations to the existing data regarding gait dysfunction in HD. Many of the studies presented have recruited small sample sizes, and results are difficult to compare due to the employment of varying technologies and protocols. In addition, though there is evidence for gait dysfunction arising even in the premanifest stage of HD, longitudinal data are lacking. Ongoing longitudinal studies, such as ENROLL-HD, may provide an ideal context to further expand on the existing knowledge. In addition to its potential as a marker of disease progression and possible endpoint in intervention studies, gait dysfunction in HD impacts overall function and quality of life. Addressing this important symptom utilizing a multidisciplinary team approach would have a significant impact on this patient population.

## Author Contributions

LT and AH contributed to the conception of the manuscript. LT wrote the first draft of the manuscript. Both authors contributed to manuscript revision, read, and approved the submitted version.

## Conflict of Interest

The authors declare that the research was conducted in the absence of any commercial or financial relationships that could be construed as a potential conflict of interest.

## Publisher’s Note

All claims expressed in this article are solely those of the authors and do not necessarily represent those of their affiliated organizations, or those of the publisher, the editors and the reviewers. Any product that may be evaluated in this article, or claim that may be made by its manufacturer, is not guaranteed or endorsed by the publisher.
